# The clinical characteristics and microbiological investigation of pediatric burn patients with wound infections in a tertiary hospital in Ningbo, China: A ten-year retrospective study

**DOI:** 10.3389/fmicb.2022.1034099

**Published:** 2023-01-09

**Authors:** Yefang Ke, Lina Ye, Pan Zhu, Zhe Zhu

**Affiliations:** ^1^Department of Clinical Laboratory, Ningbo Women and Children’s Hospital, Ningbo, Zhejiang, China; ^2^Neonatal Intensive Care Unit, Ningbo Women and Children’s Hospital, Ningbo, Zhejiang, China; ^3^Department of Blood Transfusion, HwaMei Hospital, University of Chinese Academy of Sciences, Ningbo, Zhejiang, China

**Keywords:** burn, pediatric, pathogenic, microbiological, drug resistance

## Abstract

Burn is one of the leading causes of death and disability in children worldwide, and wound infection is an excellent challenge in burn treatment. We performed a retrospective review of pediatric burn patients with wound infections to reveal their clinical data and investigate pathogens’ distribution and drug resistance patterns to provide references for treatment. As a result, 330 pediatric burn patients with wound infections were identified; 65.8% (217/330) were < 2 years old. Most of the injuries were scalded and involved <10% total body surface area in size (TBSA), mainly causing II-degree burn and II + III-degree burn. Three hundred and fifty nine strains of pathogens were isolated, the primary pathogens were *Staphylococcus aureus* (45.4%) and *Pseudomonas aeruginosa* (18.7%). Both *S. aureus* and *P. aeruginosa* isolated from 2012 to 2016 were more likely to be multi-resistant than those isolated from 2017 to 2021, as they were significantly associated with resistance to ≥4 Clinical and Laboratory Standard Institute (CLSI) classes (*p* = 0.040 and 0.006, respectively). In conclusion, children aged <2 years old were the main pediatric burn patients with wound infections. The primary bacteria isolated from the wound were *S. aureus* and *P. aeruginosa*, with a decreasing tendency of multi-resistance.

## Introduction

Burn injury is a leading cause of death and disability in the pediatric population worldwide, especially in low- and middle-income countries (Forjuoh, 2006; Haagsma et al., 2016). The loss of the skin barrier and immunosuppression caused by burn injury make patients susceptible to bacterial infection (Williams and Lee, 2021). Despite continual advances in treating burns, wound infection remains a considerable threat to burn patients.

Infectious diseases have become the primary cause of mortality in pediatric and adult burn patients (D'Abbondanza and Shahrokhi, 2021; Williams and Lee, 2021). Immediately after a burn occurs, the gut, skin, nasopharyngeal tract flora, and environmental microorganisms may reach the wound (Church et al., 2006). They may even enter the bloodstream, potentially leading to sepsis (Weaver Jr. et al., 2019). In addition, controlling and preventing infections among burn patients remains challenging. One of the most significant challenges is bacterial resistance to conventional antibiotics. The multidrug-resistant (MDR) bacteria exhibit co-resistance to many classes of antibiotics, accounting for the bulk of deaths due to sepsis (van Langeveld et al., 2017). Moreover, there was a limitation on antibiotic usage in children.

Information on the drug resistance of pathogens in the local pediatric burn unit is scarce. To better understand this disorder and provide references for treatment, we collected and analyzed the epidemiological characteristics of pediatric burn patients with wound infection and the isolated pathogenic microorganism. Additionally, new interventions for preventing nosocomial infection have been implemented since early 2017, including living far from each other, emphasizing hand hygiene, and limiting antibiotic usage. Therefore, we also compared the bacterial epidemiological data between 2012–2016 and 2017–2021 years.

## Materials and methods

### Study design and patient selection

This retrospective study was conducted at Ningbo Women and Children’s Hospital, the only pediatric tertiary hospital in Ningbo, Zhejiang, China. The institute ethics Committee approved this study (Certificate No.EC2022-M004), and the informed consent was waived.

Pediatric burn patients with pathogens isolated from their burn wounds from January 2012 to December 2021 were enrolled in this study. We excluded patients aged >14 years old, hospital length of stay (LOS) <3 days, or without complete clinical or microbiological data. Burn injury refers to those whose skin or other tissues are injured by heat, cold, ultraviolet radiation, electricity, friction, or chemicals, while most injuries in this study are due to heat from hot liquids or flame.

Demographic data, clinical characteristics (percentage of total body surface area burned [%TBSA], the severity of burn injury, and etiology), and microbiological information (isolated pathogens, antibiotic resistance) were collected from their medical records.

### Microbiology methods

Microbial cultures and antimicrobial susceptibility tests were performed when they had signs of systemic or local infection, such as fever, erythema, local warmth, serous exudate, discoloration of granulation tissue, foul odor, etc. Samples were collected with sterilized swabs from the burn wounds. Only the first positive culture results were included.

The VITEK 2 COMPACT automatic analysis system (BioMérieux, France) was used to identify pathogens and perform antimicrobial susceptibility tests. Standard strains (ATCC700323, ATCC25922, ATCC700327, ATCC29213) were taken to control the test quality.

Antibiotic resistance was defined as resistant minimum inhibitory concentration. We determined the number of isolates that were fully susceptible and the number resistant to 1 class, 2 classes, 3 classes, and ≥ 4 classes. The susceptibility breakpoints were those recommended by the Clinical and Laboratory Standards Institute guidelines (CLSI) for isolates obtained subsequently. The breakpoint criteria of the day in the analysis system were used to interpret the AST results, and the breakpoint criteria were updated promptly when the CLSI guidelines changed.

### Statistical analysis

Categorical variables were presented as percentages. The comparison between 2012–2016 isolates and 2017–2021 isolates was analyzed by logistic regression or Fisher’s exact test. A *p* < 0.05 was considered statistically significant. All statistical analyzes were performed using SPSS statistical 22.0 software (IBM, Armonk, NY, United States).

## Results

### Demographic and clinical characteristics

During the 10-year study period, 330 pediatric burn patients with wound infections were identified at our hospital. Of the total children included, 61.8% (204/330) were male, and 38.2% (126/330) were female; the male/female ratio was 1.62:1. Most of them (217/330, 65.8%) were < 2 years old. The leading cause of burns was hot water (83.3%). Most injuries were < 10% TBSA in size (245/330, 74.2%). II-degree burn (136/330, 41.2%) and II + III-degree burn (157/330, 47.6%) were the main categories depending on their severity (Table 1).

**Table 1:** Demographic and clinical characteristics of 330 burn wound infection cases.

Characteristic	*n* (%)
**Sex**	
Male	204 (61.8)
Female	126 (38.2)
**Age**	
<2 years	217 (65.8)
≥2 years	113 (34.2)
**Burn type**	
Hot water scald	275 (83.3)
Food/beverage scald	34 (10.3)
Flame	11 (3.3)
Other	10 (3.0)
**TBSA (%)**	
<10	245 (74.2)
10 ~ 19	57 (17.3)
20 ~ 29	19 (5.8)
≥30	9 (2.7)
**The severity of burn injury**	
II-degree burns	136 (41.2)
II + III-degree burns	157 (47.6)
III-degree burns	37 (11.2)
Total	330 (100)

### Pathogenic microorganism information

A total of 359 strains of pathogens were isolated from 330 children during the study period. Gram-positive bacteria, Gram-negative bacteria, and fungi accounted for 62.4% (224/359), 37.0% (133/359), and 0.6% (2/359), respectively.

Most burn wound infections were caused by a single organism (92.4%). The primary pathogens were *Staphylococcus aureus* (45.4%) and *Pseudomonas aeruginosa* (18.7%). *S. aureus* was more isolated than *P. aeruginosa* during 2012–2021, and their annual changes of the detection rate were presented as Supplementary Figure S1. The others were *Klebsiella pneumonia* (3.1%), *Enterobacter cloacae* (2.5%), *Acinetobacter baumannii* (2.5%), etc., (Table 2).

**Table 2:** Information on pathogenic microorganisms.

Variables	*n* (%)
**Infection type**	
Single Organism	305 (92.4)
Multiple Organisms	25 (7.6)
Total	330 (100)
**Bacteria**	
*Staphylococcus aureus*	163 (45.4)
*Pseudomonas aeruginosa*	67 (18.7)
*Klebsiella pneumoniae*	11 (3.1)
*Enterobacter cloacae*	9 (2.5)
*Acinetobacter baumannii*	9 (2.5)
Others	100 (27.9)
Total	359 (100)

### Antibiotic susceptibility

As *S. aureus* and *P. aeruginosa* were the two leading bacteria isolated, we analyzed their antibiotic resistance profiles detailly.

Most of the 163 *S. aureus* isolates were resistant to Penicillin (95.7%), and 46.6% (76/163) of them were methicillin-resistant *S. aureus* (MRSA). On the other hand, they were all susceptible to Linezolid, Vancomycin, and Nitrofurantoin. The *S. aureus* isolated from 2012 to 2016 had higher resistance rates to TMP-SMX, Erythromycin, and Tetracycline than those isolated from 2017 to 2021 (*p* < 0.05 in all cases; Table 3).

**Table 3:** Antibiotic resistance of *Staphylococcus aureus* among 2012–2016 isolates and 2017–2021 isolates.

Antibiotic resistance type^a^	All isolates (*n* = 163)	2012–2016 isolates (*n* = 92)	2017–2021 isolates (*n* = 71)	*p* value	OR (95%CI)
**Agents**					
Oxacillin	76 (46.6%)	44 (47.8%)	32 (45.1%)	0.727	1.117 (0.600–2.079)
TMP-SMX	62 (38.0%)	49 (53.3%)	13 (18.3%)	<0.001	4.254 (2.109–8.582)
Erythromycin	105 (64.4%)	66 (71.7%)	39 (54.9%)	0.027	2.083 (1.086–3.996)
Rifampicin	3 (1.8%)	1 (1.1%)	2 (2.8%)	0.432	0.379 (0.034–4.267)
Linezolid	0 (0.0%)	0 (0.0%)	0 (0.0%)	-	-
Clindamycin	90 (55.2%)	56 (60.9%)	34 (47.9%)	0.099	1.693 (0.905–3.166)
Moxifloxacin	18 (11.0%)	9 (9.8%)	9 (12.7%)	0.560	0.747 (0.280–1.992)
Penicillin	156 (95.7%)	90 (97.8%)	66 (93.0%)	0.150	3.409 (0.642–18.115)
Gentamicin	31 (19.0%)	19 (20.7%)	12 (16.9%)	0.546	1.280 (0.575–2.848)
Tetracycline	50 (30.7%)	36 (39.1%)	14 (19.7%)	0.009	2.617 (1.275–5.372)
Vancomycin	0 (0.0%)	0 (0.0%)	0 (0.0%)	-	-
Levofloxacin	30 (18.4%)	19 (20.7%)	11 (15.5%)	0.401	1.420 (0.627–3.215)
Nitrofurantoin	0 (0.0%)	0 (0.0%)	0 (0.0%)	-	-
**Patterns**					
Class level^b^					
0 class (no resistant)	6 (3.7%)	2 (2.2%)	4 (5.6%)	Reference	
1 class	35 (21.5%)	10 (10.9%)	25 (35.2%)	0.813	1.250 (0.197–7.942)
2 classes	22 (13.5%)	13 (14.1%)	9 (12.7%)	0.273	0.346 (0.052–2.310)
3 classes	40 (24.5%)	21 (22.8%)	19 (26.8%)	0.390	0.452 (0.074–2.757)
≥ 4 classes	60 (36.8%)	46 (50.0%)	14 (19.7%)	0.040	0.152 (0.025–0.920)

*P* value indicated the comparison between 2012–2016 isolates and 2017–2021 isolates. ^a^Resistant to single antimicrobials, 1 class, 2 classes, 3 classes, and ≥ 4 classes, resistance was defined as a resistant minimum inhibitory concentration. ^b^Based on 163 *S. aureus* isolates with complete information for all the tested antibiotics.

According to susceptibility patterns, *S. aureus* that was isolated from 2012 to 2016 were more likely to be multi-resistant, as they were significantly associated with resistance to ≥4 CLSI classes (*p* = 0.040, OR:0.152, 95% CI: 0.025–0.920; (Table 3).

Of the *P. aeruginosa*, the resistance rate to Gentamicin was highest (68.7%, 46/67), followed by Amikacin (65.7%, 44/67) and Imipenem (62.7%, 42/67). The *P. aeruginosa* isolated from 2012 to 2016 had higher resistance rates to Amikacin, Cefepime, Gentamicin, Ceftazidime, Imipenem, and Piperacillin/tazobactam than those isolated from 2017 to 2021 (*p* < 0.05 in all cases). On the other hand, the *P. aeruginosa* isolated from 2012 to 2016 had lower resistance rates to Ciprofloxacin and Levofloxacin than those isolated from 2017 to 2021; however, they did not reach significant differences (*p* = 0.05; Table 4).

**Table 4:** Antibiotic resistance of *Pseudomonas aeruginosa* among 2012–2016 isolates and 2017–2021 isolates.

Antibiotic resistance type^a^	All isolates (*n* = 67)	2012–2016 isolates (*n* = 48)	2017–2021 isolates (*n* = 19)	*P* value	OR (95%CI)
**Agents**					
Amikacin	44 (65.7%)	37 (77.1%)	7 (36.8%)	0.003	5.766 (1.826–18.207)
Ciprofloxacin	14 (20.9%)	7 (14.6%)	7 (36.8%)	0.05	0.293 (0.086–1.001)
Cefepime	36 (53.7%)	33 (68.8%)	3 (15.8%)	<0.001	11.733 (2.964–46.448)
Gentamicin	46 (68.7%)	38 (79.2%)	8 (42.1%)	0.005	5.225 (1.660–16.445)
Ceftazidime	19 (28.4%)	19 (39.6%)	0 (0.0%)	<0.001	-
Imipenem	42 (62.7%)	35 (72.9%)	7 (36.8%)	0.008	4.615 (1.493–14.270)
Levofloxacin	14 (20.9%)	7 (14.6%)	7 (36.8%)	0.05	0.293 (0.086–1.001)
Piperacillin/tazobactam	29 (43.3%)	26 (54.2%)	3 (15.8%)	0.008	6.303 (1.622–24.498)
**Patterns**					
Class level^b^					
0 class (no resistant)	20 (29.9%)	10 (20.8%)	10 (52.6%)	Reference	
1 class	3 (4.5%)	1 (2.1%)	2 (63.2%)	0.595	2.000 (0.155–25.755)
2 classes	2 (3.0%)	1 (2.1%)	1 (5.3%)	1.000	1.000 (0.055–18.304)
3 classes	15 (22.4%)	12 (25.0%)	3 (15.8%)	0.078	0.250 (0.054–1.165)
≥ 4 classes	27 (40.3%)	24 (50.0%)	3 (15.8%)	0.006	0.125 (0.028–0.553)

*p* value indicated the comparison between 2012–2016 isolates and 2017–2021 isolates. ^a^Resistant to single antimicrobials, 1 class, 2 classes, 3 classes, and ≥ 4 classes, resistance was defined as a resistant minimum inhibitory concentration. ^b^Based on 67 *Pseudomonas aeruginosa* isolates with complete information for all the tested antibiotics.

According to susceptibility patterns, *P. aeruginosa* that was isolated from 2012–2016 were more likely to be multi-resistant, as they were significantly associated with resistance to ≥4 CLSI classes (*p* = 0.006, OR: 0.125, 95% CI: 0.028–0.553; (Table 4).

Besides, the annual changes of drug resistance rate and resistance to ≥4 CLSI classes of *S. aureus* and *P. aeruginosa* were presented in Supplementary Table S1; Supplementary Figure S2. Generally, there were down trends of both *S. aureus* and *P. aeruginosa* in annual resistance to ≥4 CLSI classes from 2012 to 2021 (*p* = 0.017 and 0.146, respectively).

## Discussion

Previous studies have shown that most pediatric burn patients were < 2 years old, and boys occupied the majority (Li et al., 2021; Ruan et al., 2021; Sarginson et al., 2021). So did those pediatric burn patients with wound infections in this study. Of the 330 pediatric burn patients with wound infections, more than half (65.8%) were < 2 years old, and the boy/girl ratio was 1.62:1. Children aged <2 years old, especially boys, were very active without self-protective ability. In addition, they may be ignored by family members sometimes, leading them to be the main groups injured by burning. Besides, scald was considered as the first cause of pediatric burns in many former reports (Li et al., 2021; Loos et al., 2021; Ruan et al., 2021; Sarginson et al., 2021). Our study confirmed that hot water and food/beverage scald accounted for 83.3 and 10.3%, respectively. These remind us to strengthen child care, to protect them from hot water and food/beverage, especially for young boys. Luckily, similar to former reports, most burn children were moderate according to TBSA and severity (Armstrong et al., 2021; Ruan et al., 2021; Sarginson et al., 2021).

The infection was a great challenge for burn treatment, which led to increased pain and delayed healing, scarring, sepsis risk, hospital stays, and healthcare costs (Jeschke et al., 2020; Partain et al., 2020; Krasnoff et al., 2021). The burn wound was the central part to be infected. Identifying the primary bacterium and their drug-resistant profiles was critical for controlling the infection.

Studies have shown that *S. aureus* and *P. aeruginosa* were the leading bacteria isolated from burn wounds (Rashid et al., 2019; Mater et al., 2020; Basit et al., 2021; Cato et al., 2021; Hashemzadeh et al., 2022). While most of these studies investigated adults with/without children, little information was available on pediatric burn patients. Despite the study participants being different from the formers (Rashid et al., 2019; Mater et al., 2020; Basit et al., 2021; Cato et al., 2021; Hashemzadeh et al., 2022), the primary pathogenic bacteria detected from the burn wound of pediatric patients were also *S. aureus* and *P. aeruginosa* in the present study. In other words, *Staphylococcus* and *P. aeruginosa* were the primary bacteria infected burn wounds that are irrelevant to the host’s age. *Staphylococcus* is the resident flora in the skin (Kazmierczak and Szewczyk, 2004). *P. aeruginosa* often exists in the hospital environment (Thi et al., 2020), which may lead them to be the primary bacterium isolated from burn wounds.

The pattern of bacterial resistance is essential for epidemiology and clinical therapy. In recent years, MDR has become a global health concern (van Langeveld et al., 2017). Previous studies have reported that MRSA was the most frequent microbial agent of the outbreak and significantly increased morbidity and mortality. The rate of MRSA in the present study was 46.6%, which was higher than that reported in India (Joy et al., 2020), Saudi Arabia (Mater et al., 2020), and Morocco (Frikh et al., 2018). Fortunately, all *S. aureus* in this study were sensitive to Linezolid and Vancomycin, and the rate of resistant ≥4 CLSI classes isolates was significantly decreased. Meanwhile, the antimicrobial resistance of *P. aeruginosa* was reduced for decades. It was in accordance with a study performed in southwest China, which found that the rate of MDR *P. aeruginosa* sharply decreased in the burn-intensive units (Gong et al., 2021). In the present study, the decreasing antibiotic resistance rates of main pathogenic bacteria may mainly attribute to new interventions for preventing nosocomial infection, including living far from each other, emphasizing hand hygiene, and limiting antibiotic usage.

However, this study had some limitations. First, this was a single-center retrospective study. Second, only the first positive culture results were included in this study; therefore, it could not reflect the dynamic changes in pathogen resistance over time and after antibiotic treatment. Third, the tendency of antimicrobial resistance of *S. aureus* and *P. aeruginosa* were not intensively studied; further studies should focus on their molecular mechanisms. Therefore, a prospective study with dynamic and genetic analysis was warranted.

In conclusion, this study provided information on the clinical and microbiological data of pediatric burn patients with wound infection. Most pediatric burn patients with burn wound infection were aged <2 years old. The leading cause of burns was scalded and involved <10% TBSA, mainly causing II-degree burn and II + III-degree burn. *S. aureus* and *P. aeruginosa* were the primary bacteria isolated from burn wounds and showed a decreasing prevalence of antimicrobial resistance. Infection-control strategies and treatment should be performed according to the characteristics of pediatric burn patients and bacteria isolated.

## Data availability statement

The raw data supporting the conclusions of this article will be made available by the authors, without undue reservation.

## Ethics statement

The studies involving human participants were reviewed and approved by Ningbo Women and Children’s Hospital Ethics Committee. Written informed consent from the participants’ legal guardian/next of kin was not required to participate in this study in accordance with the national legislation and the institutional requirements.

## Author contributions

YK analyzed the data and wrote the manuscript. LY and PZ collected the data. ZZ designed the study and revised the manuscript. All authors contributed to the article and approved the submitted version.

## Funding

This study was supported by the Ningbo Public Service Technology Foundation, China (no. 2021S155) and the Medical Scientific Research Foundation of Zhejiang Province, China (no. 2022KY1158).

## Conflict of interest

The authors declare that the research was conducted in the absence of any commercial or financial relationships that could be construed as a potential conflict of interest.

## Publisher’s note

All claims expressed in this article are solely those of the authors and do not necessarily represent those of their affiliated organizations, or those of the publisher, the editors and the reviewers. Any product that may be evaluated in this article, or claim that may be made by its manufacturer, is not guaranteed or endorsed by the publisher.

## Supplementary material

The Supplementary material for this article can be found online at: https://www.frontiersin.org/articles/10.3389/fmicb.2022.1034099/full#supplementary-material
