# Supplementary material for: The clinical characteristics and microbiological investigation of pediatric burn patients with wound infections in a tertiary hospital in Ningbo, China: A ten-year retrospective study
Source: Front Microbiol. 2023 Jan 9;13:1034099. doi: 10.3389/fmicb.2022.1034099 (PMC9868836; doi:10.3389/fmicb.2022.1034099)
Supplement: Supplementary file 1 [file Table_1.DOCX]

Supplementary Table 1. Annual changes of drug resistance rate of *Staphylococcus* aureus and *Pseudomonas aeruginosa*.

| **Antibiotic** | **2012** | **2013** | **2014** | **2015** | **2016** | **2017** | **2018** | **2019** | **2020** | **2021** |
| --- | --- | --- | --- | --- | --- | --- | --- | --- | --- | --- |
| *Staphylococcus aureus* | | | | | | | | | | |
| Oxacillin | 36.4 | 60.0 | 62.5 | 44.4 | 30.8 | 9.1 | 50.0 | 37.5 | 60.0 | 71.4 |
| TMP-SMX | 100.0 | 100.0 | 33.3 | 11.1 | 15.4 | 0.0 | 22.7 | 18.8 | 13.3 | 42.9 |
| Erythromycin | 77.3 | 53.3 | 75.0 | 66.7 | 84.6 | 36.4 | 72.7 | 50.0 | 40.0 | 71.4 |
| [Rifampicin](javascript:;) | 4.5 | 0.0 | 0.0 | 0.0 | 0.0 | 0.0 | 0.0 | 0.0 | 6.7 | 14.3 |
| [Linezolid](javascript:;) | 0.0 | 0.0 | 0.0 | 0.0 | 0.0 | 0.0 | 0.0 | 0.0 | 0.0 | 0.0 |
| Clindamycin | 40.9 | 53.3 | 75.0 | 61.1 | 76.9 | 27.3 | 72.7 | 50.0 | 26.7 | 42.9 |
| [Moxifloxacin](javascript:;) | 13.6 | 20.0 | 12.5 | 0.0 | 0.0 | 0.0 | 9.1 | 12.5 | 20.0 | 28.6 |
| Penicillin | 100.0 | 100.0 | 95.8 | 94.4 | 100.0 | 90.9 | 100.0 | 81.3 | 93.3 | 100.0 |
| [Gentamicin](javascript:;) | 27.3 | 20.0 | 33.3 | 11.1 | 0.0 | 0.0 | 18.2 | 18.8 | 20.0 | 28.6 |
| [Tetracycline](javascript:;) | 40.9 | 33.3 | 50.0 | 38.9 | 23.1 | 0.0 | 18.2 | 25.0 | 26.7 | 28.6 |
| Vancomycin | 0.0 | 0.0 | 0.0 | 0.0 | 0.0 | 0.0 | 0.0 | 0.0 | 0.0 | 0.0 |
| Levofloxacin | 36.4 | 20.0 | 25.0 | 11.1 | 0.0 | 0.0 | 18.2 | 12.5 | 20.0 | 28.6 |
| Nitrofurantoin | 0.0 | 0.0 | 0.0 | 0.0 | 0.0 | 0.0 | 0.0 | 0.0 | 0.0 | 0.0 |
| *Pseudomonas aeruginosa* | | | | | | | | | | |
| [Amikacin](javascript:;) | 84.2 | 91.7 | 60.0 | 100.0 | 40.0 | 0.0 | 20.0 | 100.0 | 66.7 | 0.0 |
| Ciprofloxacin | 15.8 | 0.0 | 10.0 | 100.0 | 20.0 | 0.0 | 0.0 | 100.0 | 100.0 | 0.0 |
| Cefepime | 73.7 | 83.3 | 60.0 | 100.0 | 20.0 | 0.0 | 0.0 | 75.0 | 0.0 | 0.0 |
| [Gentamicin](javascript:;) | 89.5 | 91.7 | 60.0 | 100.0 | 40.0 | 0.0 | 40.0 | 100.0 | 66.7 | 0.0 |
| Ceftazidime | 52.6 | 66.7 | 10.0 | 0.0 | 0.0 | 0.0 | 0.0 | 0.0 | 0.0 | 0.0 |
| Imipenem | 84.2 | 75.0 | 60.0 | 100.0 | 40.0 | 0.0 | 20.0 | 100.0 | 66.7 | 0.0 |
| Levofloxacin | 15.8 | 0.0 | 10.0 | 100.0 | 20.0 | 0.0 | 0.0 | 100.0 | 100.0 | 0.0 |
| Piperacillin/tazobactam | 52.6 | 50.0 | 60.0 | 100.0 | 40.0 | 0.0 | 0.0 | 75.0 | 0.0 | 0.0 |

Data were presented as percentage.





Supplementary Figure 1. Annual changes of detection rate of *Staphylococcus* aureus and *Pseudomonas aeruginosa*.





Supplementary Figure 2. Annual changes of drug resistance rate (≥4 classes) of *Staphylococcus* aureus and *Pseudomonas aeruginosa*.
